# Stiff Substrates Enhance Endothelial Oxidative Stress in Response to Protein Kinase C Activation

**DOI:** 10.1155/2019/6578492

**Published:** 2019-04-14

**Authors:** Rebecca Lownes Urbano, Swathi Swaminathan, Alisa Morss Clyne

**Affiliations:** ^1^Mechanical Engineering and Mechanics, Drexel University, Philadelphia, PA, USA; ^2^Biomedical Engineering, Science and Health Systems, Drexel University, Philadelphia, PA, USA

## Abstract

Arterial stiffness, which increases with aging and hypertension, is an independent cardiovascular risk factor. While stiffer substrates are known to affect single endothelial cell morphology and migration, the effect of substrate stiffness on endothelial monolayer function is less understood. The objective of this study was to determine if substrate stiffness increased endothelial monolayer reactive oxygen species (ROS) in response to protein kinase C (PKC) activation and if this oxidative stress then impacted adherens junction integrity. Porcine aortic endothelial cells were cultured on varied stiffness polyacrylamide gels and treated with phorbol 12-myristate 13-acetate (PMA), which stimulates PKC and ROS without increasing actinomyosin contractility. PMA-treated endothelial cells on stiffer substrates increased ROS and adherens junction loss without increased contractility. ROS scavengers abrogated PMA effects on cell-cell junctions, with a more profound effect in cells on stiffer substrates. Finally, endothelial cells in aortae from elastin haploinsufficient mice (*Eln+/-*), which were stiffer than aortae from wild-type mice, showed decreased VE-cadherin colocalization with peripheral actin following PMA treatment. These data suggest that oxidative stress may be enhanced in endothelial cells in stiffer vessels, which could contribute to the association between arterial stiffness and cardiovascular disease.

## 1. Introduction

Due to the highly mechanical nature of the cardiovascular system, cardiovascular disease has long been accepted as both a biomechanical and biochemical disease. Arterial stiffness, which increases with hypertension and aging among others, is an independent predictor of cardiovascular risk [1–3]. Arteries reversibly stiffen when smooth muscle cells contract and irreversibly stiffen as elastin is degraded and collagen increases [4–9]. Stiff arteries have long been known to contribute to cardiovascular mortality by increasing cardiac afterload [10]; more recently, stiff arteries have also been shown to contribute to endothelial dysfunction, an initiating step in atherosclerosis [11–13]. *In vitro*, endothelial monolayers on stiff polyacrylamide (PA) gels were more permeable [12, 14, 15]. In animal models, endothelial permeability was elevated in stiffened aortae from older mice [12]. Mesenteric arteries from elastin haploinsufficient (*Eln*+/-) mice had enhanced angiotensin-induced vasoconstriction and impaired endothelium-dependent vasodilation [13], although aortae from these same animals do not [16]. In human subjects, endothelial flow-mediated vasodilation was inversely correlated with aortic stiffness [17, 18]. Thus, arterial stiffness alone may contribute to cardiovascular risk by altering critical endothelial functions.

However, cardiovascular risk factors rarely occur in isolation but rather cluster in certain individuals. Little is known about how arterial stiffness interacts with other cardiovascular risk factors such as diabetes and inflammation. Both hyperglycemia and inflammatory cytokines such as tumor necrosis factor-*α* (TNF-*α*) increase endothelial cell oxidative stress due to increased production and decreased scavenging of reactive oxygen species (ROS). Elevated ROS have been implicated in both hypertension and atherosclerosis pathogenesis [19]. ROS are a family of highly reactive oxygen-containing molecules, including superoxide (O_2_^−^), hydrogen peroxide (H_2_O_2_), hydroxyl radical (^·^OH), and peroxynitrite (ONOO^−^), which play an important role in many signaling pathways, such as cell proliferation, survival, and metabolism [20]. Superoxide is produced by the mitochondrial electron transport chain, as well as through protein kinase C- (PKC-) induced NADPH oxidase upregulation and activation [21–23]. NADPH oxidase assembly at the cell membrane requires Rac, which is enhanced by substrate stiffness [24].

The effect of arterial stiffness on endothelial ROS production in response to an external stimulus such as hyperglycemia or TNF-*α* has not yet been investigated. However, these risk factors also activate many other endothelial cell signaling pathways. Therefore, to isolate substrate stiffness effects on endothelial ROS production, we used phorbol 12-myristate 13-acetate (PMA), a PKC activator widely used to stimulate ROS production *in vitro*. In previous studies, PMA treatment increased endothelial monolayer permeability but did not increase actinomyosin contractility, as measured by silicon substrate wrinkling, myosin light chain (MLC) phosphorylation, or MLC kinase activation [25, 26]. Phorbol esters may instead induce barrier loss through intermediate filament or actin cytoskeleton reorganization [27–29]. Thus, PMA enables investigation of substrate stiffness effects on ROS production without stimulating actinomyosin contractility.

We hypothesized that stiff substrates would increase endothelial ROS in response to PMA, resulting in actin fiber formation and cell-cell junction loss. We used varied stiffness PA gels to study PMA-induced endothelial ROS, actin fiber formation, and adherens junction loss. Abdominal aortae from wild-type (WT) and *Eln*+/- mice were treated with PMA ex vivo and imaged *en face*. We now show that substrate stiffness enhances PMA-induced oxidative stress in endothelial monolayers *in vitro* and alters actin fiber reorganization and adherens junction morphology both *in vitro* and ex vivo.

## 2. Materials and Methods

### 2.1. Animals

All experiments were performed according to protocols approved by the Drexel University College of Medicine Animal Studies Committee. *Eln*+/- mice were generated as previously described [30]. 8-12-week-old WT and *Eln*+/- mice of both sexes, backcrossed several generations into the C57BL/6 background (Charles River), were used. All mice were genotyped to confirm elastin heterozygosity, and decreased elastin lamellae thickness was confirmed in select animals by immunohistochemistry. Mice were provided access to food and water *ad libitum* at 22°C and a 12-hour light/dark cycle.

### 2.2. Atomic Force Microscopy (AFM)

AFM was used to quantify aortic stiffness in wild-type (WT) and *Eln*+/- mouse aortae. The aorta was dissected and transferred to ice-cold HEPES buffer (140 mM NaCl, 5 mM KCl, 1 mM CaCl_2_, 1.2 mM MgSO_4_, 1.2 mM Na_2_HPO_4_, 10 mM HEPES, 10 mM sodium acetate, and 5 mM glucose, pH 7.4). Excess tissue was cleaned from the outside of the vessel, and the vessel was cut open longitudinally to expose the endothelium. Each aorta was cut into four segments—two thoracic segments and two abdominal segments—producing four samples per aorta. Samples were carefully mounted, the endothelium facing up, on a coverslip using Loctite 401 medical grade adhesive (Henkel) and submersed in PBS. The endothelium was removed by gentle scraping with a cotton-tip applicator, based on a published protocol [31]. Subendothelial stiffness was determined by AFM using precalibrated cantilevers (spring constants between 0.10 and 0.17 N/m) with 10 *μ*m spherical tips. Between three and nine indentations were made at different locations along each sample. The force-indentation curve for each indentation was fit to the Hertz model down to 200 nm indentation using a custom MATLAB code to produce a stiffness value [32]. Subendothelial stiffness was calculated as the average of the individual stiffness values of each sample.

### 2.3. Cell Culture and Polyacrylamide (PA) Gel Sample Preparation

Primary porcine aortic endothelial cells (PAEC) were isolated by the collagenase dispersion method and cultured in low glucose Dulbecco's modified Eagle's medium (DMEM, Corning) supplemented with 5% fetal bovine serum (FBS, HyClone), 1% glutamine, and 1% penicillin-streptomycin (Invitrogen). Cells were used up to passage 9.

**Figure 1:**
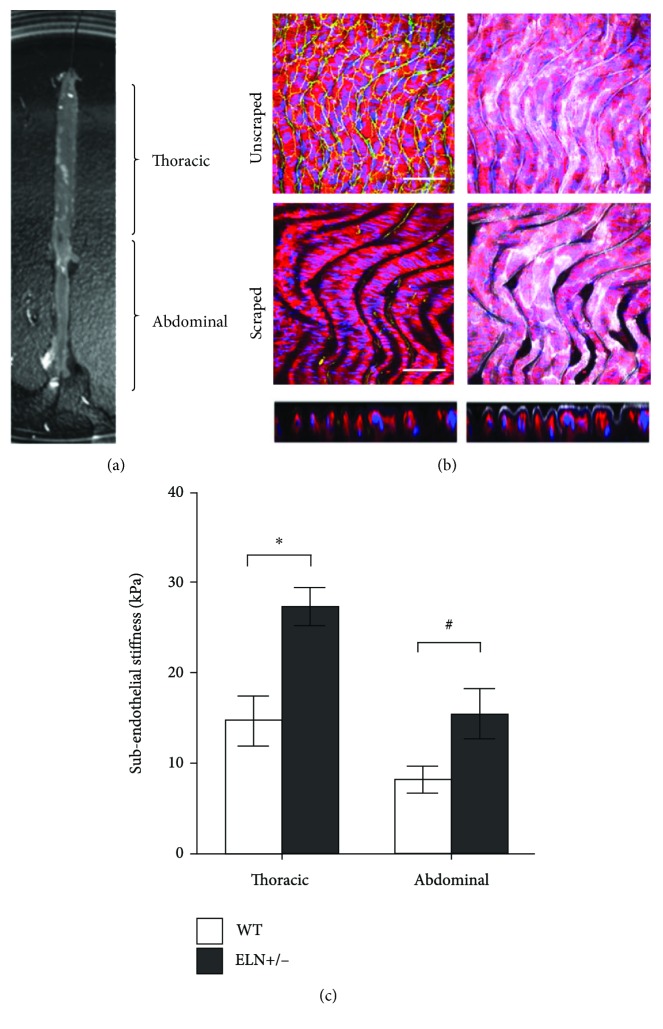
Subendothelial stiffness increased in the thoracic and abdominal aortae of Eln+/- mice. (a) Longitudinally dissected mouse aorta opened to expose the endothelial surface. (b, top) Unscraped aorta showing the intact endothelium via *β*-catenin (green), cell structure using actin (red) and nuclei (blue), and the subendothelial matrix using collagen IV (white). Artery is shown en face. (b, bottom) Scraped aorta showed that the endothelium was removed since no *β*-catenin (green) was observed. The subendothelial matrix (collagen IV, white) remained intact and contiguous both en face and in cross section (smaller images). Scale bar = 50 *μ*m. (c) Subendothelial stiffness of aortae from WT and Eln+/- mice. Thoracic and abdominal aortic sections were indented by AFM using a silicon nitride cantilever with a 10 *μ*m spherical tip to measure subendothelial stiffness (^∗^*p* < 0.01 and ^#^*p* < 0.05 by Student's *t*-test). Three aortae were tested for each condition.

6, 14, or 29 kPa was selected for the PA gel stiffnesses based on the subendothelial stiffnesses measured in WT and *Eln*+/- mouse aorta (Figure 1). PA gels were prepared following well-established protocols [33, 34]. Briefly, a bottom coverslip was made hydrophilic by consecutive incubations with 0.1 M sodium hydroxide (NaOH, Sigma-Aldrich), 3-aminopropyltrimethoxysiliane (3-APTES, Sigma-Aldrich), and 0.5% glutaraldehyde (Electron Microscopy Sciences). A top coverslip was made hydrophobic by applying SurfaSil (1,7-dichloro-octamethyltetrasiloxane, Thermo Scientific). A solution containing varying amounts of 40% acrylamide and 2% bisacrylamide (Bio-Rad) was prepared based on the desired gel stiffness (Table 1). Ammonium persulfate (Bio-Rad) and tetramethylethylenediamine (TEMED, Bio-Rad) were added to the acrylamide/bisacrylamide solution to achieve final concentrations of 0.1% *w*/*v* and 0.3% *v*/*v*, respectively, initiating gel polymerization. Polymerizing gel solution was added to the bottom coverslip, and the top coverslip was quickly inverted onto the polymerizing gel to create a flat surface. After gel formation, the top coverslip was removed. Elastic modulus was confirmed by AFM. To make the surface adhesive to cells, the gel was UV-activated using sulfo-SANPAH (Thermo Fisher) in dimethyl sulfoxide (DMSO, Fisher Scientific) and 50 mM HEPES buffer and then incubated with 100 *μ*g/mL type I collagen (BD Biosciences) at 37°C for 3 hours at room temperature or at 4°C overnight. The collagen-coated gel was rinsed in sterile phosphate-buffered saline (PBS) and UV-sterilized prior to cell seeding.

PAEC were seeded on collagen-coated PA gels in phenol red-free DMEM and cultured to confluence for three days in a growth medium. Cells were then serum-starved overnight in phenol red-free DMEM containing 1% FBS, 1% glutamine, and 1% penicillin-streptomycin. After serum starvation, cells were left untreated or treated with 1 *μ*M PMA for varying durations. In some cases, endothelial monolayers were pretreated with ROS scavengers (4 mM N-acetyl cysteine or 50 mM sodium pyruvate, Sigma) for 1 hour prior to PMA.

### 2.4. ROS Assay

ROS were measured using 5-(and-6)-chloromethyl-2′,7′-dichlorodihydrofluorescein diacetate (CM-H_2_DCFDA), which passively diffuses into cells where it is cleaved by intracellular esterases and then oxidized by ROS to yield a fluorescent adduct. 100 *μ*M tert-butyl hydroperoxide (tBHP), which produces intracellular hydrogen peroxide, was the positive control. After PMA or tBHP treatment, samples were rinsed with warmed HBSS buffer (0.137 M NaCl, 5.4 mM KCl, 0.25 mM Na_2_HPO_4_, 5.6 mM glucose, 0.44 mM KH_2_PO_4_, 1.3 mM CaCl_2_, 1.0 mM MgSO_4_, and 4.2 mM NaHCO_3_). 25 *μ*M CM-H_2_DCFDA in phenol red-free DMEM was added to each sample and incubated for 25 minutes at 37°C, protected from light. To label nuclei, bisbenzimide (0.2 *μ*g/mL, Thermo Fisher) was added to each sample for an additional 5 minutes. After thorough washing in HBSS buffer, samples were immersed in warmed phenol red-free DMEM and imaged in an Olympus Fluoview 1000 microscope as confocal *z*-stacks (1 *μ*m step size).

### 2.5. Confocal Microscopy and Image Analysis

Endothelial cells on PA gels were imaged by confocal microscopy and analyzed using MATLAB. *In vitro* cell samples were rinsed once with ice-cold PBS and fixed with ice-cold 4% paraformaldehyde. Samples were then permeabilized with 0.2% Triton X-100 in PBS for 15 minutes and blocked with 1% bovine serum albumin (BSA) in PBS for 1 hour at room temperature. After fixation, mouse aortae were simultaneously blocked and permeabilized in PBS containing 1% BSA and 0.3% Triton X-100. Endothelial cells on PA gels were labeled with primary antibodies for VE-cadherin (1 : 200, Santa Cruz), *β*-catenin (1 : 200, Thermo Fisher), or pMLC (1 : 200, Cell Signaling) in 1% BSA in PBS overnight at 4°C. After several rinses with PBS, samples were then incubated with the appropriate Alexa Fluor 488 or 633 (1 : 200, Thermo Fisher) secondary antibody, rhodamine phalloidin (16.5 nM, Invitrogen), and bisbenzimide (0.2 *μ*g/mL) for 1 hour at room temperature, protected from light. Samples were rinsed twice with PBS and mounted in 1 : 1 glycerol : PBS. Confocal *z*-stacks were acquired for all samples with either a 0.25 or 0.5 *μ*m step size (for *in vitro* and ex vivo samples, respectively) using an Olympus Fluoview 1000 confocal microscope at 60x magnification.

A custom MATLAB code was created to quantify ROS and pMLC. The background was subtracted using a 50 × 50 pixel area. Images were then binarized using the same threshold as determined using Otsu's method, which calculates a threshold based on pixel intensity distribution [35]. Noise was removed from binarized images by excluding small objects (less than 9 pixels for ROS, less than 30 pixels for pMLC). The number of remaining pixels with intensities above the threshold (“positive” pixels) was counted for each image. Three images per sample were quantified using this method and averaged to quantify ROS or pMLC in each sample.

### 2.6. PKC Activity Assay

After treatment, cells on PA gels were quickly rinsed with ice-cold PBS and inverted onto 50 *μ*L lysis buffer (20 mM MOPS, 50 mM *β*-glycerophosphate, 50 mM sodium fluoride, 1 mM sodium orthovanadate, 5 mM EGTA, 2 mM EDTA, 1% NP40, 1 mM dithiothreitol, 1 mM benzamidine, 1 mM phenylmethanesulfonyl fluoride, 10 *μ*g/mL leupeptin, and 10 *μ*g/mL aprotinin) for 10 minutes at 4°C. Lysed cells were then scraped from the gel substrates, collected in prechilled Eppendorf tubes, and centrifuged at 4°C for 15 minutes at 13,000 rpm. The supernatant was collected, and the protein concentration was determined by BCA assay (Thermo Fisher). PKC activity was quantified in control or treated cell lysates using an ELISA-based PKC kinase assay (Enzo) as per the manufacturer's instructions. Absorbance (450 nm) was measured on a microplate reader (Thermo LabSystems, Multiskan Spectrum). Relative kinase activity was calculated as follows:

(1) $$\begin{array}{c} \text{Relative kinase activity}=\frac{\left(\text{Average absorbanc}{\text{e}}_{\text{sample}}-\text{Average absorbanc}{\text{e}}_{\text{blank}}\right)}{\text{Quantity of crude protein used per assay}}. \end{array}$$

### 2.7. Statistical Analysis

All statistical analyses were conducted using MATLAB's statistical toolbox. Graphs represent mean ± standard deviation. Multiple groups were compared using either two-way or *n*-way ANOVA with post hoc Tukey-Kramer test, and two groups were compared by Student's *t*-test. Within each PKC assay, conditions were tested in duplicate. For ROS measurement, conditions were tested in triplicate. All experiments were conducted at least two times, with at least three samples per condition.

## 3. Results

### 3.1. Subendothelial Stiffness Was Higher in Eln+/- as Compared to WT Mouse Aorta

Macroscale arterial stiffness, measured by pulse wave velocity or pressure myography, increases in mice genetically engineered to produce less elastin (*Eln*+/-) [36, 37]. However, aortic stiffness had not been characterized by atomic force microscopy in this mouse model. The longitudinally dissected mouse aorta (Figure 1(a)) shows the mounting technique and the thoracic and abdominal sections. Aortae with the intact endothelium were first labeled for *β*-catenin (green) to show endothelial cell-cell junctions, actin (red) and nuclei (blue) to highlight cell structure, and collagen IV (white) to view the basement membrane (Figure 1(b), top). When we removed the endothelium from the longitudinally dissected mouse aortae, we no longer observed *β*-catenin, confirming that cells were removed. The collagen IV layer remained intact and contiguous, indicating that the subendothelial matrix remained intact (Figure 1(b), bottom; collagen IV integrity was most clear in the cross-sectional image). However, it is possible that endothelial removal did damage the subendothelial layer, as evidenced by the spaces in the fluorescently labeled samples. We therefore repeated the atomic force microscopy in both scraped and unscraped WT mouse aortae and found no difference in aortic stiffness measurements. When aortic samples were indented by AFM, the thoracic and abdominal aortic subendothelium from *Eln*+/- mice was about 1.75-fold stiffer than that of WT mice (Figure 1(c)). The thoracic aorta was consistently stiffer than the abdominal aorta in both genotypes.

### 3.2. PMA-Induced Increased Oxidative Stress in Endothelial Cells on the Stiffest PA Gels

**Figure 2:**
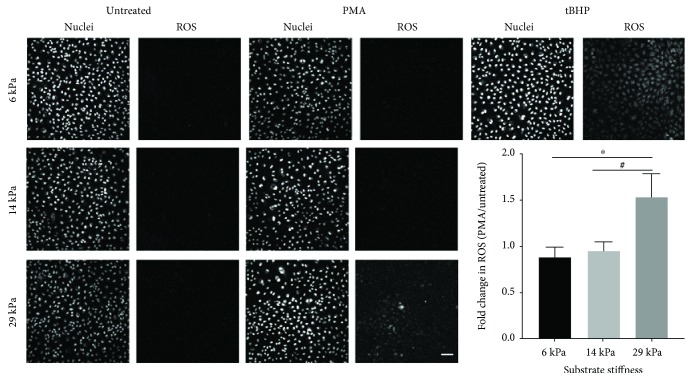
ROS increased with substrate stiffness following PMA treatment. PAEC monolayers on 6, 14, and 29 kPa gels were treated with 1 *μ*M PMA for 10 minutes. Tert-butyl hydroperoxide (tBHP) was the positive control. Cell nuclei were labeled with Hoechst, and ROS with CM-H_2_DCFDA. Samples were imaged at 20x by confocal microscopy. Scale bar is 25 *μ*m. Oxidative stress was quantified using the number of positive pixels (above the threshold) using the custom MATLAB code. PMA-treated samples were normalized to untreated samples on the same substrate stiffness. The effect of substrate stiffness was significant by one-way ANOVA (*p* < 0.01). ^#^*p* < 0.05 and ^∗^*p* < 0.01 by post hoc Tukey-Kramer test.

We then created 6, 14, and 29 kPa PA gels, which correspond to aortic stiffnesses in WT and *Eln+/-* mice, to determine if different ROS levels were produced by PMA-treated endothelial monolayers cultured on substrates of varied stiffness. Each sample was treated with 1 *μ*M PMA for 10 minutes, based on preliminary experiments showing maximum cell viability and ROS at this dose and time, and consistently imaged by confocal microscopy. ROS were statistically similar following PMA treatment of cells on 6 and 14 kPa gels. In addition, endothelial cells on stiff substrates did not show any baseline increase in ROS. However, in endothelial cells on 29 kPa substrates that were treated with PMA, ROS increased by more than 50% (*p* < 0.01 compared to fold change in cells on 6 kPa gels, Figure 2). Substrate stiffness effects on the PMA-induced fold change in ROS were also significant by one-way ANOVA (*p* < 0.01). These results demonstrate that stiffer substrates increase endothelial ROS in response to PMA.

### 3.3. Endothelial Cell PKC Increased in Response to PMA Independent of PA Gel Stiffness

**Figure 3:**
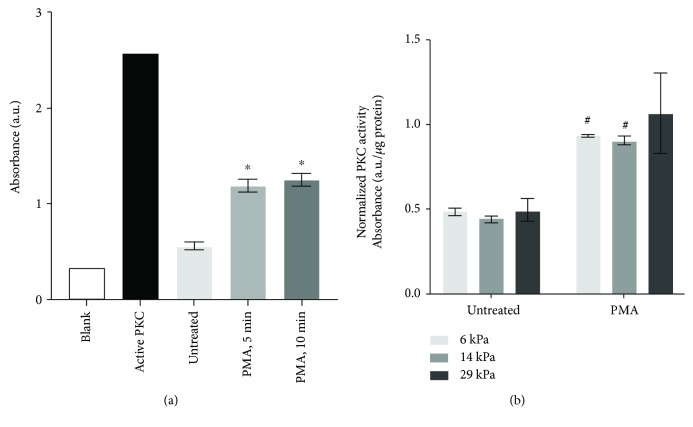
PKC activity increased with PMA independent of substrate stiffness. (a) PKC activity was measured following 1 *μ*M PMA treatment in endothelial cells on glass substrates. Purified active PKC was the positive control. ^∗^*p* < 0.01 compared to that untreated by Student's *t*-test. (b) PAEC monolayers on 6, 14, and 29 kPa substrates were treated with 1 *μ*M PMA for 5 minutes. PMA was significant by two-way ANOVA (*p* < 0.001), but substrate stiffness was not significant. ^#^*p* < 0.05 by post hoc Tukey-Kramer test compared to that untreated.

PMA induces ROS production through PKC signaling [38, 39]. We therefore measured PKC activity in PMA-treated endothelial cells on 6, 14, and 29 kPa PA gels to determine if PKC activation increased on stiffer substrates. PKC activity in PAEC increased 3-4-fold within 5 minutes of PMA treatment (Figure 3(a)). However, PKC activity in cells stimulated with PMA did not change significantly whether the cells were on soft or stiff PA gels (Figure 3(b)). Therefore, the PMA-induced differences in oxidative stress on stiffer substrates were not related to PKC activation.

### 3.4. Endothelial Cells Formed More Actin Stress Fibers in Response to PMA on Stiffer Gels

**Figure 4:**
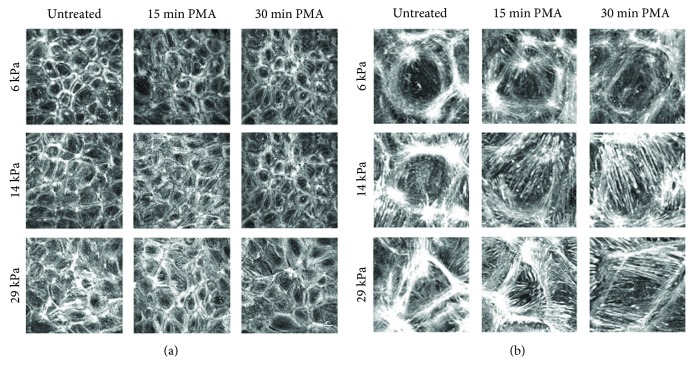
Actin stress fiber formation was greater in endothelial cells on stiffer substrates following PMA. PAEC monolayers on 6, 14, or 29 kPa gels were treated with 1 *μ*M PMA for 15 or 30 minutes prior to fixation and immunofluorescent labeling of actin (rhodamine phalloidin). (a) Maximum intensity projection from confocal *z*-stacks at 60x magnification. Scale bar is 25 *μ*m. (b) Magnified representative cells from (a).

ROS lead to endothelial actin fiber formation [40]. PMA-stimulated cells on increasing stiffness substrates were labeled for actin fibers to determine if substrate stiffness-dependent oxidative stress increased actin fiber formation. In untreated samples, actin fibers were primarily located around the cell periphery on all substrates, although the effect was more pronounced in cells on the softest 6 kPa gels (Figure 4(a), representative cell magnification in Figure 4(b)). Following 15 minutes of PMA treatment, actin fibers appeared in cells on the 14 and 29 kPa gels, but not in cells on the 6 kPa gels. This effect was even more pronounced following 30 minutes of PMA treatment, with larger stress fibers and nearly complete peripheral actin loss in endothelial cells on 14 and 29 kPa gels. Cells on 6 kPa gels largely retained peripheral actin with PMA treatment.

**Figure 5:**
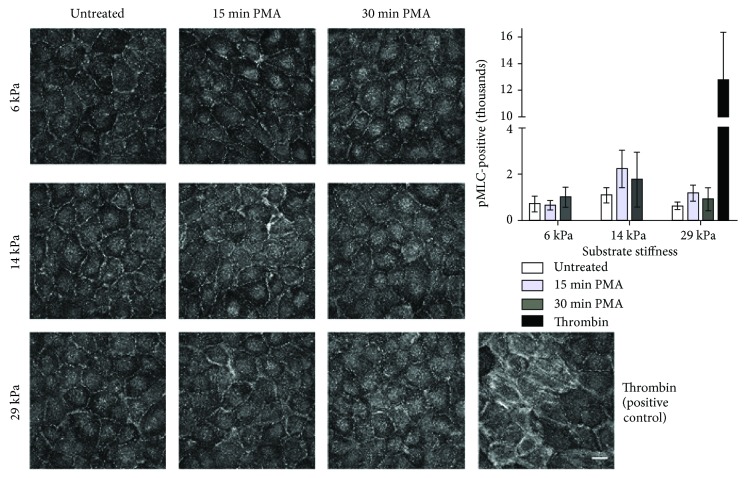
PMA treatment did not increase pMLC localization to actin stress fibers in cells on varied stiffness substrates. PAEC monolayers on 6, 14, or 29 kPa gels were treated with 1 *μ*M PMA for 15 or 30 minutes, prior to fixation and pMLC immunofluorescent labeling. For the positive control, cells on a 29 kPa gel were treated with 10 U/mL thrombin for 30 minutes. Images are maximum intensity projections from 60x confocal *z*-stacks. Scale bar is 25 *μ*m. pMLC-positive pixels were quantified using the custom MATLAB code. Stiffness and PMA treatment were not significant by *n*-way ANOVA.

PAECs were then labeled for pMLC to determine whether ROS-induced actin stress fiber formation was associated with increased actinomyosin contractility through pMLC localization to actin stress fibers. 1 *μ*M PMA treatment for 15 or 30 minutes did not induce pMLC translocation to actin fibers or increase overall pMLC (Figure 5). In contrast, the positive control (10 U/mL thrombin for 30 minutes) increased overall pMLC approximately 14-fold, with pMLC localized along the actin fibers. These results indicate that PMA-induced actin fiber formation in cells on stiffer substrate did not correspond to actinomyosin contractility.

### 3.5. Adherens Junctions Became Less Reticular in Response to PMA on Stiffer Gels

**Figure 6:**
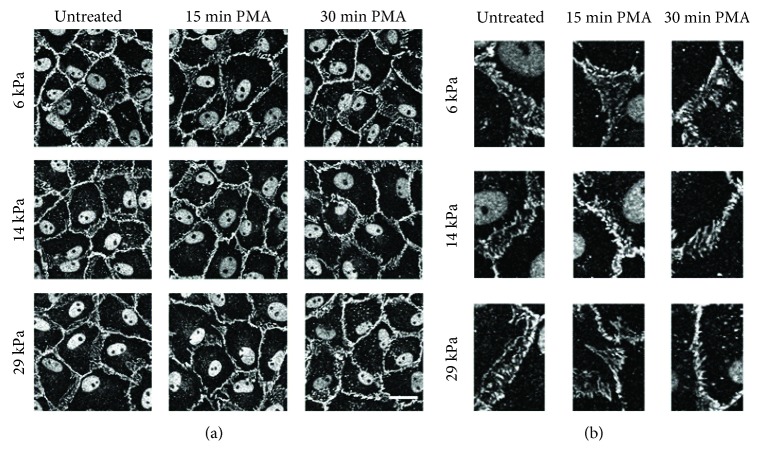
Reticular adherens junction loss was greater in cells on stiffer substrates following PMA. PAEC monolayers on 6, 14, or 29 kPa gels were treated with 1 *μ*M PMA for 15 or 30 minutes, prior to fixation and immunofluorescent labeling of the cell-cell junction protein *β*-catenin. (a) Maximum intensity projection from confocal *z*-stacks at 60x magnification. Scale bar is 25 *μ*m. (b) Representative junctions.

ROS induce cell-cell junction loss, which has been attributed in part to adherens junction protein phosphorylation and internalization [41–43]. We therefore measured if stiff substrates exacerbate ROS-mediated endothelial adherens junction loss in response to PMA. In untreated cells on 6, 14, and 29 kPa gels, wide reticular adherens junctions were evident between adjacent cells (Figure 6(a), representative magnified junctions in Figure 6(b)). In contrast to what has been observed in other published work, we did not observe any changes in adherens junction based on substrate stiffness alone perhaps due to the use of a different endothelial cell type [12]. After 15 or 30 minutes of PMA treatment, reticular junctions were mostly maintained in cells on the 6 kPa gels. In contrast, cells on the stiffest 29 kPa substrates lost most junction reticular structures and instead had linear or disrupted cell-cell junctions. These results demonstrate that endothelial reticular junction structure loss is exacerbated by stiffer substrates in response to the ROS-stimulant PMA.

**Figure 7:**
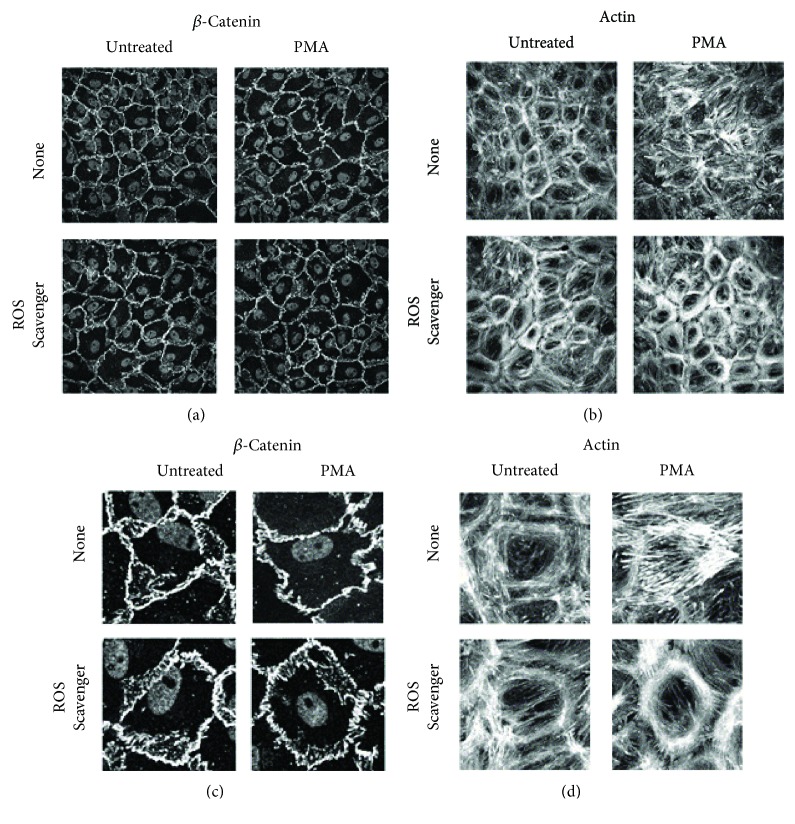
ROS scavengers prevented PMA-induced adherens junction loss and actin fiber redistribution. PAEC monolayers were pretreated with ROS scavengers (4 mM N-acetyl cysteine, 50 mM sodium pyruvate) for 1 hour before the 30-minute treatment with 1 *μ*M PMA. Samples were fixed and immunofluorescently labeled for (a) *β*-catenin, with representative cells magnified in (c), and immunofluorescently labeled for (b) actin, with representative cells magnified in (d). Images are maximum intensity projections from 60x confocal *z*-stacks. Scale bar is 25 *μ*m.

To confirm that ROS were responsible for the PMA-induced changes in adherens junctions and actin fiber formation, the ROS scavengers N-acetyl cysteine and sodium pyruvate were administered for 1 hour prior to PMA treatment. These experiments were performed on glass coverslips, since junction loss following PMA exposure was highest on these stiffest substrates. ROS scavengers themselves did not affect cell-cell junction structure, and cells treated with PMA alone showed linear and invaginated adherens junctions (Figure 7(a), representative cell magnification in Figure 7(c)). In PAEC pretreated with ROS scavengers prior to PMA, the junction morphology change was abrogated. More strikingly, ROS scavengers prevented PMA-induced actin reorganization (Figure 7(b), representative cell magnification in Figure 7(d)). Cells treated with the ROS scavengers prior to PMA showed peripheral actin bands which were similar to those in untreated cells. Thus, oxidative stress was likely responsible for PMA-induced reticular junction loss and actin fiber formation.

## 4. Discussion

Oxidative stress and more specifically the enzyme responsible for superoxide production, NADPH oxidase, have been implicated in cardiovascular disease and atherosclerosis in particular [44, 45]. We now show that stiffer substrates exacerbate endothelial cell oxidative stress. In response to PMA, endothelial cells on the stiffest substrates showed more ROS and actin stress fibers and showed greater adherens junction loss, which was not attributed to cell contractility. Stiffer aortas from *Eln*+/- mice also showed less VE-cadherin at cell-cell membranes and increased peripheral actin fiber formation in response to PMA. Since PMA-induced PKC activation was not affected by substrate stiffness, it is likely that substrate stiffness affected cells through alternative pathways. These data suggest that oxidative stress and its detrimental downstream effects on endothelial cells and vascular disease may be enhanced in stiffer arteries.

The vascular mechanics of the elastin haploinsufficient mouse have been extensively studied, both in terms of passive mechanical stretch in response to increasing pressure and in terms of vasodilation and constriction in response to biochemical stimuli [13, 37, 46, 47]. These studies focused primarily on the decrease in total elastin in the vascular wall, as well as the increase in elastin lamellae. We and others did not find any changes in other extracellular matrix proteins, in particular collagen, which is the other primary protein thought to define vascular wall stiffness. However, some recent studies in other tissues in elastin haploinsufficient mice suggest that there are also collagen changes in these animals. The lungs of Eln +/- mice contained nearly twice as much collagen 1 and lysyl oxidase, an important collagen crosslinker, as WT mice [48]. Achilles tendons of Eln+/- mice had the same total collagen content as WT mice but different collagen fibril diameter distribution [49]. Thus, it is possible that the increased stiffness we measured in the Eln+/- aorta relates to changes in collagen content and/or structure.

The thoracic aorta was consistently stiffer than the abdominal aorta in both WT and Eln+/- mice. These data agree with human studies in which aortic stiffness decreased with distance from the heart, especially in older patients [50, 51]. Other studies in C57BL/6 mice demonstrated that the aortic elastic modulus was highest in the distal thoracic aorta and lowest in the abdominal aorta [52]. In a subsequent study, it was shown that the decrease in aortic stiffness along the length of the aorta was accounted for by a decrease in total and lamellar elastin [53]. Since our data show a proportionally similar decrease in aortic stiffness from the thoracic to the abdominal sections in both WT and Eln+/- mice, it is likely that elastin content is important to the regional stiffness variation.

Our data support other recently published studies showing that substrate stiffness affects not only single endothelial cells but also confluent endothelial monolayers [12]. *In vitro* studies of cell response to substrate stiffness began when Pelham and Wang first used protein-coated PA gels to show that both rat kidney epithelial and 3T3 fibroblasts spread to a greater extent on stiff than soft substrates [54]. Since that seminal paper, many cell types were shown to change their morphology [55–58], motility [59, 60], differentiation [61, 62], and proliferation [63, 64] in response to substrate stiffness. For endothelial cells specifically, single cells increase the spread area [65, 66], stiffness [67], cell-matrix and cell-cell forces [66, 68], and proliferation [69] with substrate stiffness. However, as cells proliferated and reached confluency, substrate-dependent differences were diminished or no longer observed [70, 71]. Similarly, we did not observe any changes in endothelial cell and actin stress fiber morphology, focal adhesion size, or focal adhesion number in endothelial cell monolayers on substrates of different stiffness. However, while these morphological responses to substrate stiffness are no longer observed as endothelial cells reach confluency, this study shows that both endothelial biochemical responses and cell-cell interactions do change with substrate stiffness.

ROS, specifically superoxide and its byproduct hydrogen peroxide, have been shown to regulate actin fibers in vascular cells [40, 72–74]. Actin fiber formation in subconfluent reoxygenated hypoxic aortic endothelial cells was inhibited by superoxide dismutase overexpression, suggesting a key role for superoxide [75]. Superoxide can reversibly oxidize proteins, including actin itself. In endothelial cells, actin oxidation may be essential for actin polymerization during cell migration. For example, in migrating mouse aortic endothelial cells, actin monomer incorporation into actin fibers was diminished following treatment with the NADPH oxidase inhibitor diphenyleneiodonium (DPI) and a superoxide dismutase mimetic [76]. Alternatively, superoxide can oxidize RhoA, enhancing GDP dissociation and subsequent activation. Fibroblasts with an oxidation-resistant form of RhoA did not form stress fibers in response to hydrogen peroxide [77]. While the source of increased ROS in endothelial cells on stiffer substrates remains unknown, we hypothesize that stiff substrates increase NADPH oxidase production or assembly, since NADPH oxidase appears to produce the most nonmitochondrial superoxide in endothelial cells [78]. We hope to investigate this mechanism further in future studies.

Although endothelial oxidative stress has not been studied on varied stiffness substrates, endothelial superoxide production is mechanosensitive, specifically in response to shear stress [79]. Bovine aortic endothelial cells produced three times more superoxide under oscillatory shear stress compared to laminar flow [80]. Shear stress activates Rac, which is downstream of integrin activation and contributes to ROS production [81, 82]. Epithelial cells have been shown to produce more ROS when on stiffer substrates. MMP-3-stimulated ROS production was approximately 3.5-fold higher in epithelial cells on 4.02 kPa substrates compared to 0.13 kPa substrates; *β*1 integrin subunit knockdown eliminated ROS production in response to MMP-3 [83]. These findings suggest that integrin activation-induced Rac1 activity contributes to ROS production in cells on stiffer substrates [84].

The increase in adherens junction disruption could be either contractility-dependent or contractility-independent. Permeability agents, including thrombin, lipopolysaccharide (LPS), TNF-*α*, and vascular endothelial growth factor (VEGF), activate the Rho/ROCK pathway and cell contractility [85]. The ROCK inhibitor Y-27632 prevented adherens junction disruption in endothelial monolayer studies, although some effects may be endothelial subtype-dependent (e.g., macrovascular or microvascular) [86, 87]. In epithelial cell protrusions, H_2_O_2_ increased actin polymerization, cofilin activity, and barbed ends; however, myosin IIA did not colocalize with actin fibers in H_2_O_2_-treated cell protrusions [88]. These data fit with our results that actin contractility did not increase with oxidative stress. Therefore, it is more likely in our case that oxidative stress induced contractility-independent adherens junction disruption. ROS also disrupt cell-cell junctions through VE-cadherin phosphorylation. Endothelial cell treatment with permeability agonists, such as VEGF and histamine, resulted in VE-cadherin tyrosine phosphorylation [89, 90]. In HUVECs, the ROS scavenger N-acetyl cysteine prevented VE-cadherin phosphorylation by TNF-*α* [91]. Thus, we hypothesize that adherens junction protein phosphorylation resulted in cell-cell junction loss, although we did not directly measure it.

ROS can also lead to adverse effects on the endothelium beyond adherens junction loss. For example, superoxide (O_2_^−^) interacts with nitric oxide (NO) to form peroxynitrite. This interaction effectively decreases the NO availability, which is needed for vasodilation [92, 93]. Superoxide also uncouples eNOS by oxidizing the eNOS cofactor tetrahydrobiopterin (BH_4_) [94–96]. Uncoupled eNOS produces more superoxide instead of NO [97], which further increases peroxynitrite. Protein nitration by peroxynitrite is widely observed in many cardiovascular diseases [98]. Thus, vascular stiffness-induced endothelial oxidative stress could have damaging effects beyond endothelial barrier function.

Substrate stiffness also affects other cell types beyond endothelial cells, including fibroblasts, breast cancer cells, and stem cells [99, 100]. *In vivo*, tumors are stiffer than their surrounding environment, which may alter both basal function and inflammatory response in all of these cell types. In addition, some tumors overexpress specific NADPH oxidases [101]. This overexpression could couple with increased tumor stiffness to further magnify oxidative stress in tumors. Tumor oxidative stress contributes to tissue injury and DNA damage in premalignant conditions, as well as in cancer initiation and progression. Since the tumor cells themselves may be resistant to oxidative stress, the injury to the surrounding tissue may be more severe [102]. Thus, stiffness-associated ROS inhibition could potentially decrease cancer-induced damage and inhibit cancer metastasis through compromised blood vessels.

While our work shows that PMA-induced oxidative stress increases with substrate stiffness, our research is not without limitations. Phorbol esters, including PMA, are found in certain plants and can cause toxicity in animals when consumed [38]. Yet, PMA itself is not involved in cardiovascular disease pathogenesis. We chose to use PMA to isolate ROS production from cell contractility in cell-cell junction loss [25, 26]; however, these studies should be repeated using a physiologically relevant ROS inducer (e.g., tumor necrosis factor-*α*). PA gels do not fully capture the endothelial mechanical environment, including substrate viscoelasticity and relaxation as well as shear stress or strain. Several recent papers have shown interactions between shear stress and substrate stiffness, demonstrating that softer substrates augmented the atheroprotective effects of laminar shear stress [103, 104]. We also were not able to specifically detect superoxide in intact vessels under physiological conditions due to technical challenges. In future studies, a superoxide-specific indicator such as dihydroethidium could be used along with NADPH oxidase component knockdown to support the hypothesis that stiffness-induced integrin activation enhances superoxide production by NADPH oxidase [105].

## 5. Conclusions

This work illustrates a novel potential mechanism for substrate-enhanced oxidative stress in response to PKC activation in the endothelium. Since many endothelial cell studies are performed on tissue culture polystyrene of essentially infinite stiffness, these studies may overestimate endothelial cell response to stressors. Further study of the interaction between arterial stiffness and oxidative stress could improve therapies to prevent or ameliorate endothelial barrier dysfunction.

## Figures and Tables

**Table 1:** Acrylamide and bisacrylamide concentrations used to create varying elastic modulus PA gels.

Acrylamide	Bisacrylamide	Elastic modulus
7.5%	0.05%	6 kPa
10%	0.1%	14 kPa
10%	0.3%	29 kPa

## Data Availability

AFM indentation curves, PKC quantification, and immunofluorescence images used to support the finding of this study may be released upon application to the corresponding author.
